# Experimental data and computational modeling link auxin gradient and development in the *Arabidopsis* root

**DOI:** 10.3389/fpls.2014.00328

**Published:** 2014-07-07

**Authors:** Natalie M. Clark, Maria A. de Luis Balaguer, Rosangela Sozzani

**Affiliations:** Department of Plant and Microbial Biology, North Carolina State University, RaleighNC, USA

**Keywords:** *Arabidopsis*, auxin gradient, root development, stem cell niche, computational modeling

## Abstract

The presence of an auxin gradient in the *Arabidopsis* root is crucial for proper root development and importantly, for stem cell niche (SCN) maintenance. Subsequently, developmental pathways in the root SCN regulate the formation of the auxin gradient. Combinations of experimental data and computational modeling enable the identification of pathways involved in establishing and maintaining the auxin gradient. We describe how the predictive power of these computational models is used to find how genes and their interactions tightly control the formation of an auxin maximum in the SCN. In addition, we highlight known connections between signaling pathways involving auxin and controlling patterning and development in *Arabidopsis*.

## INTRODUCTION

Hormones are a crucial component of plant developmental pathways, affecting processes such as embryogenesis, growth, organ size, and stress response (Wolters and Jurgens, 2009). Among these hormones is auxin (most frequently found as indole-3-acetic acid, IAA), which contributes to the development, organization, and patterning of the entire plant (Vanneste and Friml, 2009). Specifically, auxin controls the organization and patterning of the root, including the stem cell niche (SCN; Overvoorde et al., 2010). In *Arabidopsis*, most auxin biosynthesis occurs in tryptophan-dependent pathways, although other pathways have been discovered (for more details about auxin biosynthesis, refer to Tivendale et al., 2014). Once synthesized in the shoot, auxin travels through the root via a number of polar auxin transporters (Bennett et al., 1996; Swarup et al., 2001; Petrasek et al., 2006; Wisniewska et al., 2006). This polar auxin transport results in a gradient of auxin from the shoot with a maximum in the root SCN. This gradient and maximum have been shown to affect root development and patterning (Berleth et al., 2007; Grieneisen et al., 2007; Laskowski et al., 2008). In this review, we first examine how auxin transport proteins and local auxin biosynthesis in particular areas of the root contribute to the gradient and maximum formation. We then address pathways downstream of auxin that affect root patterning, specification of the SCN, and lateral root development. Mathematical modeling is used to predict and eventually validate the proposed models along with experimental data. Finally, we will also discuss these mathematical models to better understand the link between auxin and development.

## AUXIN GRADIENT AND MAXIMUM FORMATION IN THE ROOT SCN

**FIGURE 1:**
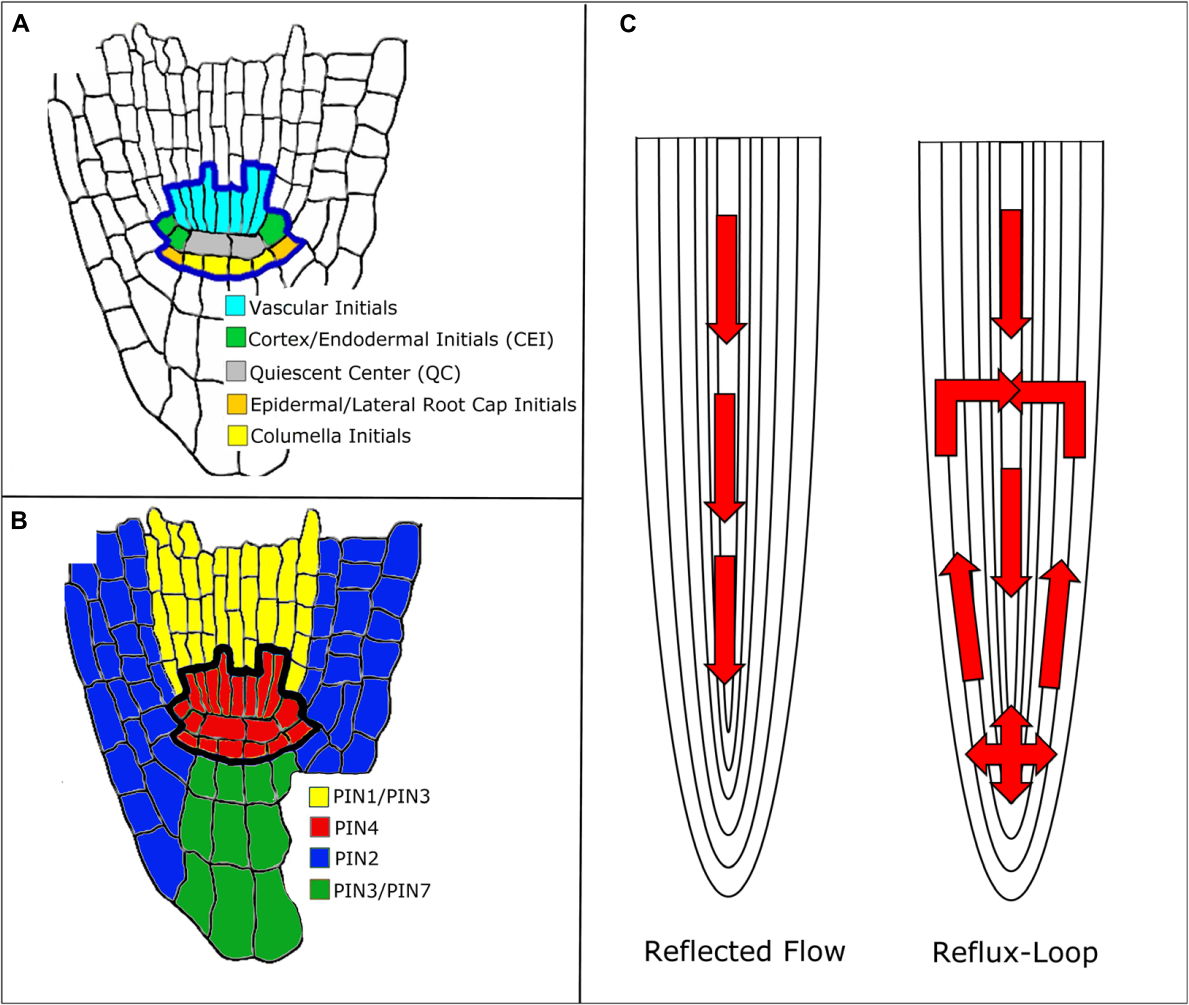
**Mechanisms of auxin transport in the *Arabidopsis* root. (A)** Root stem cell niche (SCN) organization. **(B)** PIN protein localization around the SCN. Note that the colors do not represent the exact localization of the PIN protein at the membrane, rather the cell populations where the PIN proteins are located. **(C)** Reflected flow (Mironova et al., 2010) and reflux-loop (Grieneisen et al., 2007) mechanisms of auxin transport.

In *Arabidopsis*, some auxin is synthesized in the shoot (Cheng et al., 2006). Once synthesized, auxin travels from the shoot down to the root tip, where it forms a maximum in the SCN (Grieneisen et al., 2007; **Figure 1A**). The SCN consists of the quiescent center (QC) surrounded by four types of stem cells. The QC cells are relatively mitotically inactive and produce cellular signals that regulate the maintenance of the stem cells around them (Dolan et al., 1993; Sozzani and Iyer-Pascuzzi, 2014). Auxin transport throughout the root is facilitated by influx and eﬄux carriers. The AUXIN1/LIKE-AUX1 (AUX1/LAX) transporters are the major influx carriers (Bennett et al., 1996; Swarup et al., 2001) while PIN FORMED (PIN) protein family members are the major auxin eﬄux carriers (Petrasek et al., 2006; Wisniewska et al., 2006; Kleine-Vehn and Friml, 2008). In *Arabidopsis*, there are eight known PIN proteins: PIN1–PIN8 (Blilou et al., 2005; Mravec et al., 2009; Ding et al., 2012). PIN5, PIN6, and PIN8 belong to a less characterized subclade of PIN proteins and are localized in the endoplasmic reticulum membrane (Mravec et al., 2009). PIN1 is localized mainly at the basal end of vasculature cells. PIN2 is located apically (the end of the cell closest to the shoot) in epidermal and lateral root cap cells and basally in cortical cells. PIN3 is expressed in the columella, at the basal side of vasculature cells, and at the lateral side of the pericycle cells of the elongation zone. PIN4 is localized in the SCN and basally in provascular cells. Finally, PIN7 resides at lateral and basal membranes of provascular cells in the meristem and elongation zone, whereas in the columella cells it coincides with the PIN3 domain. (**Figure 1B**; Blilou et al., 2005). These various PIN proteins produce diverse auxin flux patterns. Since they are located in different cells, this results in different auxin transport rates throughout the root (Blilou et al., 2005). Thus, PIN localization is crucial for the formation of the auxin gradient and maximum (Grieneisen et al., 2007).

Two mechanisms have been proposed to explain how PINs establish and maintain the auxin gradient: the reflux-loop mechanism (Grieneisen et al., 2007) and the reflected flow mechanism (Mironova et al., 2010; **Figure 1C**). The reflected flow mechanism only considers PIN1 protein localization and, instead of considering each individual cell population, generalizes to vasculature and non-vasculature cells. PIN1 transports auxin down the shoot into the SCN. Once the auxin maximum passes a certain threshold, PIN1 proteins degrade, preventing the transport of auxin down the root and forming the auxin gradient. The reflected flow model is based on the activator–inhibitor mechanism. In this mechanism, pattern formation is induced by both a positive and negative regulator. In the reflected flow model, auxin serves as both the activator and inhibitor of PIN1 protein expression (Mironova et al., 2010). Grieneisen et al. (2007) built upon the reflected flow mechanism by adding PIN2 and PIN3 to the model. In their reflux-loop mechanism, auxin is transported from the vasculature to the root tip. Once the auxin reaches the SCN, PINs transport auxin laterally and then up the root, back to the vasculature. Unlike other models, the reflux-loop mechanism accounts for the spatial structure of cells. This allows diffusion and cell permeability to be described as two separate parameters instead of one combined flux parameter. Additionally this model accounts for specific sizes and shapes of individual cells (Grieneisen et al., 2007). In order to show that their proposed mechanisms produce the auxin gradient and maximum, both of these groups used mathematical modeling. The reflected flow model is a set of ordinary differential equations (ODEs) that measure the change in auxin and PIN protein concentrations over time (Mironova et al., 2010). The reflux-loop model is a set of partial differential equations that measure the change in auxin concentrations over time and space (between the cells of the root). Unlike in the reflected-flow model, the reflux-loop model assumes that PIN transport of auxin is constant (Grieneisen et al., 2012). Both groups then produced computer simulations of their models that illustrate how the equations produce both the auxin gradient and maximum in the SCN (Mironova et al., 2010; Grieneisen et al., 2012). Since both of these methods are able to generate the auxin gradient and maximum, one might suggest that they cooperate together to regulate root development. Indeed, a model that combines both of these mechanisms shows how they regulate root apical meristem (RAM) development. Regeneration of the RAM is provided by the reflected flow mechanism while the reverse fountain enables RAM maintenance (Mironova et al., 2012). Crucially, these models depend on a particular PIN localization: the PIN proteins must be located in a specific cell and in a specific orientation. However, the exact distribution of PIN proteins within the root is uncertain since the concentrations and distribution of PIN proteins within the cell can change over time. Auxin is known to regulate PIN distribution, but it is unclear whether auxin concentration, flux, or both contribute to PIN localization (for more on PIN distribution, see Goh et al., 2014). Recently, Tian et al. (2014) adapted the reflux-loop model (Grieneisen et al., 2007 to account for experimentally observed PIN localization (Kleine-Vehn and Friml, 2008). This adapted model cannot produce the auxin maximum in the QC, suggesting that there are other pathways besides PIN-mediated auxin transport that maintain the auxin maximum (Tian et al., 2014).

Another contributing factor to the auxin maximum is the synthesis of auxin in the QC within the SCN of the root (Brady et al., 2007; Stepanova et al., 2008; Petersson et al., 2009). Auxin influx within the root tip is regulated by AUX1, which has been shown to control which tissues have high auxin levels (Band et al., 2014). The addition of local auxin synthesis and AUX1 expression in the root tip improves the previous PIN transport models. In this mathematical model, the auxin maximum in the QC depends on AUX1 expression in the lateral root cap and not on the specific PIN localization in the epidermis (Band et al., 2014). Moreover, auxin production and transport within the root tip is a distinct process from auxin production and transport from the shoot. Overproduction of auxin in the shoot cannot rescue auxin deficiency in the root tip (Chen et al., 2014). Thus, transport of auxin through AUX1 as well as local synthesis of auxin in the root tip contributes to the formation of the auxin gradient and maximum.

One important component of these mathematical models is the measurement of auxin levels within the *Arabidopsis* root. Both the *DR5* promoter and the DII-VENUS sensor can be used as proxies for auxin content. The* DR5* promoter drives expression of a reporter gene, such as β-glucuronidase (GUS) that allows one to measure auxin production and distribution (Ulmasov et al., 1997; Sabatini et al., 1999). The auxin-interaction domain DII drives the fast maturing yellow fluorescent protein VENUS. Unlike *DR5*, DII-VENUS rapidly degrades in the presence of auxin (Brunoud et al., 2012). Importantly, these two auxin reporters have different expression patterns: while *DR5* expression fits the traditional auxin gradient with a maximum in the QC, DII-VENUS expression suggests that there are cell type-specific differences in auxin distribution (Ulmasov et al., 1997; Sabatini et al., 1999; Brunoud et al., 2012). Recently, an auxin-responsive transcriptomic analysis found that auxin response genes fit one of two categories: either the genes are characterized by high expression in the root tip with peaks toward the shoot, or the genes have a graded meristematic response. While the first of these groups fits the stereotypical auxin gradient, the second suggests that there could be other cell type-specific auxin gradients (Bargmann et al., 2013). In the future, it would be interesting to study how the addition of multiple auxin gradients affects the previously developed mathematical models.

It is clear that the formation of the auxin gradient and maximum does not depend on one pathway but on a number of pathways. Auxin is produced both in the shoot and in the SCN. The PIN proteins are responsible for transporting auxin from the shoot down to the SCN, while AUX1 transports auxin throughout the SCN. The key to determining that all of these mechanisms are necessary is the use of mathematical and computational modeling. When the mathematical models for the PIN transport pathway were unable to generate the auxin maximum under all conditions, new models were developed to tie in AUX1-mediated transport and auxin production in the root tip. The next logical step is to use this same mathematical modeling approach to address auxin’s role in root development.

## AUXIN ROLE IN ACD MECHANISMS AND ROOT DEVELOPMENT

The pathways in which auxin has been reported to be involved occur in a wide range of developmental processes, including the embryonic and postembryonic SCN specification and maintenance, and the initiation and growth of new organs as lateral root initiation and development (Casimiro et al., 2001; Marchant et al., 2002; Laskowski et al., 2008; Swarup et al., 2008; Yoshida et al., 2014). Auxin gradient, decreasing from the QC toward the transition zone (TZ -between the apical meristem and basal elongation region), plays a role in controlling meristem patterning and maintenance. In particular, auxin acts with another hormone, cytokinin, to ensure a balance between cell division, promoted by auxin (Blilou et al., 2005) and cell differentiation, promoted by cytokinin (Ioio et al., 2008). Through the upregulation of the expression of tyrosylprotein sulfotransferases (TPSTs), auxin also affects growth factors in *Arabidopsis*, such as phytosulfokine peptides and root meristem growth factors (RGFs; Zhou et al., 2010). The auxin maximum and gradient contribute to positioning asymmetric cell divisions (ACDs) in a specific tissue layer in the root (Cruz-Ramírez et al., 2012) and are related to SCN specification (Sabatini et al., 1999, 2003; Benjamins and Scheres, 2008; Dinneny and Benfey, 2008; Zhou et al., 2010). Mechanisms such as those involved in ACDs positioning and meristem maintenance often have reciprocal influences on the formation of auxin maximum and gradient (Cruz-Ramírez et al., 2012; Moubayidin et al., 2013; Tian et al., 2014). Computational modeling is a powerful tool, necessary to test complex biological hypotheses, that allows an understanding of the interactions among the elements involved in the pathways (Grieneisen and Scheres, 2009). It also allows us to merge knowledge gained from different mechanisms, facilitating our understanding of the connections between multiple pathways involved in development (Narula et al., 2013). In this section, we review some root development mechanisms involving auxin, with a special focus on those influencing the SCN. We highlight new root development mechanisms involving auxin where mathematical and/or computational modeling has been integrated (Laskowski et al., 2008; Jones et al., 2009; Cruz-Ramírez et al., 2012; Tian et al., 2014).

The role of auxin in root stem cell specification in zygotic embryogenesis has long been known (Friml et al., 2003; Weijers et al., 2005; Breuninger et al., 2008). Local auxin synthesis in the SCN regulates PIN1 polarization and the auxin maximum, resulting in proper cell polarization during embryogenesis (Robert et al., 2013; Wabnik et al., 2013). Auxin activates feedback repressors of cytokinin signaling, the *ARABIDOPSIS* RESPONSE REGULATOR genes ARR7 and ARR15 (Müller and Sheen, 2008). Loss of function of these genes in the basal cell during early embryogenesis results in a defective root stem cells, indicating that the interaction between auxin and cytokinin is critical for specifying root stem cell niche (Müller and Sheen, 2008). It was more recently shown that auxin influx carriers play a role in patterning of the embryonic root (Ugartechea-Chirino et al., 2010). The results in this study indicated redundancy within the AUX1 LAX family in the establishment of cell organization in the radicle apex of arabidopsis.

During post-embryonic development, auxin is also related to SCN positioning, patterning and specification (Sabatini et al., 1999, 2003; Zhou et al., 2010). Specifically, auxin stimulates the transcription of genes PLETHORA1 (PLT1) and PLT2, which encode members of the AP2 class of transcription factors (Aida et al., 2004). These genes are critical for stem cell specification and maintenance, from the basal embryo to the embryonic root up to post-embryonic root meristem (Aida et al., 2004). PLTs are necessary for root formation and for mediating the developmental response to auxin in the root (Galinha et al., 2007). PLTs act in an interaction network with PIN proteins to control patterning and growth of the root and to stabilize the position of the SCN (Blilou et al., 2005; Dinneny and Benfey, 2008). Auxin upregulates expression of the *Arabidopsis* TPST gene (Zhou et al., 2010), which affects expression of PLT1, PLT2, PIN genes, and auxin biosynthetic genes, and is believed to maintain root SCN through the root meristem growth factor RGF1 (Matsuzaki et al., 2010). Loss of TPST function leads to defects in the root SCN and root growth and development (Komori et al., 2009; Zhou et al., 2010), thus linking auxin, PINs, PLTs, and TPST acting in a postembryonic SCN maintenance mechanism.

Previous experimental observations had concluded that PIN proteins are localized at the apical side of epidermis cells and to the basal-outer lateral side of cortex cells (Kleine-Vehn and Friml, 2008; Wabnik et al., 2011). Tian et al. (2014) incorporated these observations into a simplified version of the reflux-loop root computer model (Grieneisen et al., 2007), which resulted in a model that could not reproduce the auxin accumulation in the QC cells as observed experimentally (Tian et al., 2014). This suggested additional mechanisms in auxin maxima localization. In addition to auxin, the transcription factor WUSCHEL-related homeobox 5 (WOX5) is also believed to participate in SCN maintenance (Sarkar et al., 2007). Subsequent experimental observations and computational simulations by Tian et al. (2014) indicated that WOX5 modulates expression of auxin biosynthetic genes in the QC. It was also found that this is balanced through the activity of IAA17 auxin response repressor (Tian et al., 2014). A loop that connects auxin and WOX5, the WOX5–IAA17 feedback circuit, was thus proposed. The WOX5–IAA17 loop was shown to contribute to the maintenance of auxin maximal responses in the QC cells, and, in turn, to affect the patterning of the root SCN (Tian et al., 2014). Specifically, the predictive model developed by Tian et al. (2014) aimed to describe the contribution of the WOX5–IAA17 feedback circuit to the maintenance of auxin maximal responses in the QC. The model consists of a cellular grid tissue template, generated with the VV (Vertex–Vertex) programming language (Smith et al., 2006), and a set of coupled ODEs. The model predicted (1) a WOX5 expression maximum in the QC cells, which contributed to auxin production and maintenance of auxin maximum in these cells and (2) the decrease of auxin concentration from the QC toward distal columella cells. These computational simulations matched the experimental results, which indicated that the WOX5–IAA17 circuit might be responsible for auxin maximum maintenance in the root tip and in turn for root stem cell differentiation. The predictions made by the model thus, were observed experimentally.

**FIGURE 2:**
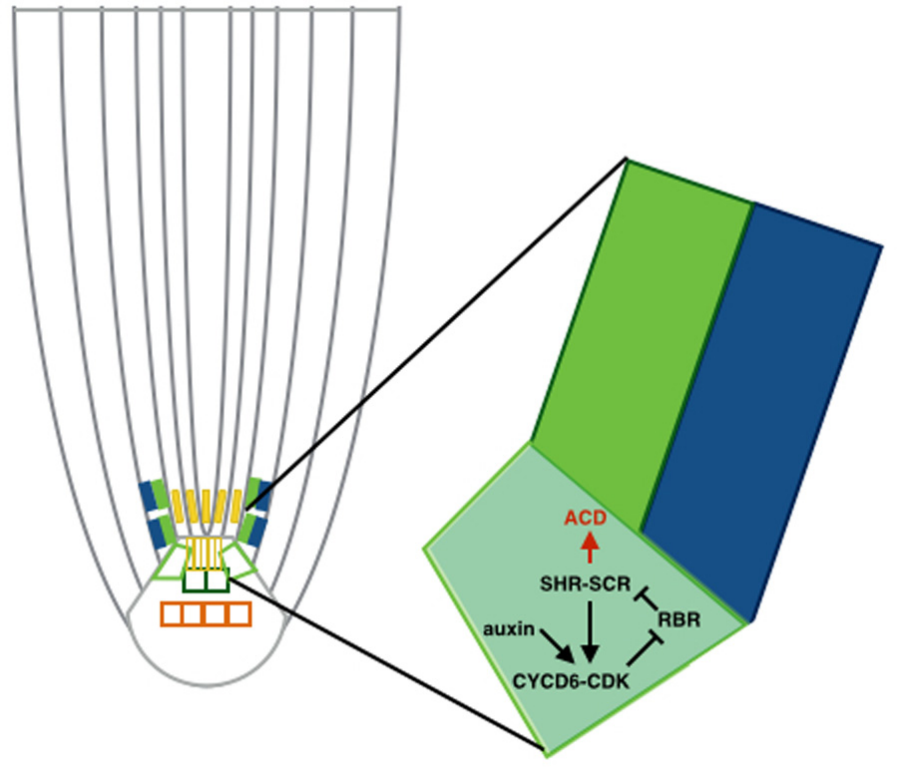
**Asymmetric cell divisions (ACD) regulated by auxin maximum.** Schematic of a cortex-endodermis initial cell, where auxin positively regulates transcription of CYCD6;1 and consequently triggers the ACD. The auxin maximum in the QC restricts the ACD to the SCN.

This auxin maximum in the QC was also shown to regulate localization of the ACDs along with the SHORTROOT-SCARECROW (SHR-SCR) pathway (Cruz-Ramírez et al., 2012; see **Figure 2**). Specifically, the RETINOBLASTOMA-RELATED (RBR) protein binds the stem cell regulator SCR. RBR is phosphorylated through a CYCLIND6;1-CDK complex, which hinders SCR activity. CYCD6;1 is at the same time transcriptionally regulated by a complex containing SCR and SHR, which creates a positive feedback loop. Auxin maximum positively regulates transcription of CYCD6;1 as well, constraining the ACDs to the SCN. Cruz-Ramírez et al. (2012) developed a predictive model to unravel this mechanism, revealing the existence of a double positive feedback loop that grants this system with a switch-like ACD behavior. The model describes a nested feedback circuit representing the spatiotemporal control of ACDs, integrating auxin-mediated tissue polarity in the longitudinal developmental axis (Grieneisen et al., 2007) with the SHR expression pattern in the radial developmental axis. The model consists of a set of coupled ODEs that capture the dynamics of the key components within each cell, which are in turn integrated within the tissue context. Through a combination of this model and experimental data, the authors show that auxin and SHR influx trigger the ACD state of the bistable circuit and the radial confinement of SHR. All the predictions made by the model are consistent with the data from this study.

In addition to regulating localization of the ACD mechanism within the SHR-SCR pathway, SCR has been shown to be responsible for other root developmental aspects. SCR in the QC cells controls auxin production, thus governing cell division of the surrounding stem cells and, at the same time, the rate of cell differentiation of meristematic daughter cells at the TZ (Moubayidin et al., 2013). Moubayidin et al. (2013) proposed a descriptive model that, based on their observations, reflected the dependence of root meristem activity on SCR, although no mathematical description was provided. The model particularly shows how SCR directly suppresses the cytokinin-response transcription factor ARR1 in the QC cells, thus titrating local auxin production and stem cell division. Auxin produced in the QC, via polar auxin transport machinery, activates ARR1 at the transition zone, thus committing meristematic cells to differentiate. This model proposes a single pivotal gene, SCR, which orchestrates a reciprocal cross talk between cytokinin and auxin. This mechanism contributes to form the meristem zonation along the apical-basal axes, coordinating cell division with cell differentiation at the opposite ends of root meristem, thus leading to a correct root organ growth.

Another role for cytokinin and auxin in the SCN specification was recently elucidated (Zhang et al., 2013). Cytokinin adjusts auxin distribution in the RAM via the auxin influx and eﬄux carriers, PINs and AUX/LAX, consequently regulating the mitotic activity in the QC (Zhang et al., 2013). Specifically, the authors showed that cytokinin affects the auxin response in the QC via downregulation of LAX2, in turn negatively regulating QC specification.

There is also evidence of roles for auxin in root development outside the SCN. For example, auxin has been related to lateral root initiation and development (Xie et al., 2000; Casimiro et al., 2001; Marchant et al., 2002; Swarup et al., 2008; De Smet et al., 2010), and the crosstalk between auxin and cytokinin is involved in radial pattern of the root, vascular differentiation, root gravitropism, and together with ethylene, lateral root initiation (Aloni et al., 2006). NAC1, a member of the NAC family, is induced by auxin and activates expression of two downstream auxin-responsive genes to promote lateral root development (Xie et al., 2000). It was later proposed that auxin enters cortical and epidermal cells underlying new lateral root primordia, inducing the expression of auxin influx carrier LAX3 (Swarup et al., 2008). Increased LAX3 facilitates auxin uptake, reinforcing expression of LAX3. This feedback loop results in auxin accumulation that triggers cell wall remodeling genes (Swarup et al., 2008). Other experiments showed that auxin accumulates on the outside of the vasculature in bent regions of the root (Laskowski et al., 2008). These areas correspond to AUX1 up-regulation, PIN down-regulation, and maxima of lateral root initiation regions. This suggests that auxin influx plays a major role in determining the density of root primordia (Laskowski et al., 2008). In this study, Laskowski et al. (2008) used a predictive, computational model focused on describing the dynamics of auxin transport through the root and understanding the impact of curvatures in auxin accumulation and lateral root formation. Specifically, the model included cell geometries, typical PIN distributions, changes in cell-shapes caused by curvature of the root, and auxin transport. The model was able to predict that curvature of the root induces auxin accumulation on the outside of the vasculature in bent regions, where lateral root initiation is highly likely to occur. These studies suggested that auxin was responsible for initiation of LR primordia and the spatial patterning of lateral organs in roots. Recent studies, however, suggested that both LR positioning and root bending are periodic responses that may be regulated by an oscillating gene expression mechanism (Moreno-Risueno et al., 2010; Van Norman et al., 2014). Moreno-Risueno et al. (2010) indicated that although auxin may be involved in the process, it does not seem to be sufficient to initiate prebranch sites. Instead, their data suggested that oscillating genes establish the temporal and spatial distribution of LRs along the primary root axis. These findings were later restated by Van Norman et al. (2014), where carotenoids were additionally found to be necessary for the output of the LR clock and establishment of the LR prepattern. This suggests that LR formation may be simultaneously regulated at several molecular levels, although a relationship between hormones such as auxin and the endogenous clock-like mechanism underlying LR patterning has not yet been reported.

Another important aspect of root development in which auxin is known to take part is initiation and growth of root hairs (Masucci and Schiefelbein, 1994; Pitts et al., 1998; Rahman et al., 2002; Jones et al., 2009). It has been suggested that insensitivity to ethylene affects the auxin-driven root hair elongation, placing the hormones auxin and ethylene in another crosstalk pathway that plays a role in root hair development (Rahman et al., 2002). It was later revealed that the auxin-influx transporter AUX1 is not present in root-hair cells, but it is instead highly expressed in adjacent non-hair cell files (Jones et al., 2009). Jones et al. (2009) used a three dimensional model of auxin flow in the root’s tip, which suggests that transport of auxin from non-hair cells maintains an auxin supply to hair cells during their growth. Together, these results clearly indicate the involvement of auxin and auxin transport in root hair initiation and development.

## CONCLUDING REMARKS

Recent studies have described the predicted roles of auxin in SCN specification, patterning, and maintenance, as well as its contribution to root growth, lateral root initiation, and root hair development. The reciprocal influences of pathways affecting the formation of an auxin maximum and gradient, and this gradient affecting root development, have been highlighted. Elucidating the mechanisms by which signaling pathways involving auxin and controlling patterning and development is a key challenge for understanding the systems biology of plant growth. Due to its polar movement, it is difficult to predict the auxin dynamics by studying it locally. The computational models above presented have yielded advances in our understanding of the role of auxin in development, allowing more global and intuitive interpretations of diverse data and results. Thus, the use of computational models as a tool to study and understand mechanisms underlying root development has enabled such systems-level modeling. Identifying critical features governing such regulatory networks and their integration will be the next necessary step toward a predictive mechanistic model. An accurate characterization of the quantitative parameters will therefore be essential to generate models that accurately capture the behavior of the system. This will allow us to identify and to predict decision-making signals, ultimately leading to control of plant growth and development.

## Conflict of Interest Statement

The authors declare that the research was conducted in the absence of any commercial or financial relationships that could be construed as a potential conflict of interest.
